# A Smartphone-Based Ecological Momentary Intervention for Workplace Mental Health: Randomized Controlled Trial

**DOI:** 10.2196/72435

**Published:** 2026-09-08

**Authors:** Sisi Li, Lik Hang Lincoln Lo, Chiu Yi Charlie Lau, Megan Lam, Meanne Chan

**Affiliations:** 1 Department of Community Health and Behavioral Medicine, School of Public Health, Shanghai Jiao Tong University School of Medicine, Shanghai, China; 2 Wofoo Joseph Lee Consulting and Counselling Psychology Research Centre, Lingnan University, Tuen Mun, China (Hong Kong); 3 Neurum Health, Central Hong Kong Island, China (Hong Kong); 4 Department of Population Health and Policy, School of Health and Medical Sciences, City St. George’s, University of London, Cranmer Terrace, London, SW17 0RE, United Kingdom, +44 (0) 2086729944

**Keywords:** workplace stress, mobile health, mHealth, online intervention, ecological momentary intervention, EMI, ecological momentary assessment, EMA, digital intervention

## Abstract

**Background:**

Work-related stress has been widely associated with an increased risk of various mental disorders and poor mental well-being. The fast-growing mobile health services industry has provided new opportunities for workplace mental health.

**Objective:**

This randomized controlled trial examined the effectiveness of *Neurum* (Neurum Limited), a smartphone-based intervention tool featuring ecological momentary assessments and interventions that aims to reduce workplace stress in real-time and real-world settings.

**Methods:**

A total of 201 working adults were recruited for a 4-week smartphone-based intervention that incorporated cognitive behavioral therapy, mindfulness exercises, and self-regulation exercises delivered on *Neurum*. A simple randomization procedure was used. Participants in the intervention group were encouraged to log mood journals, complete mental health exercises, and provide user feedback whenever applicable. The key outcome was measured by the Depression, Anxiety, and Stress Scale-21 items (DASS-21; Cronbach α=0.87).

**Results:**

The intervention group consisted of 102 participants, while the control group consisted of 99 participants. More participants dropped out from the intervention group (n=21) than from the control group (n=2; *χ*^2^_1_=18.259; *P*<.001). The final sample consisted of 178 participants (male: 85/178, 47.8%; female: 93/178, 52.2%; mean age of 34.65, SD 7.67 y). Analyses revealed that after the 4-week intervention, the DASS-21 scores decreased in the intervention group (mean difference [MD]_post-pre intervention_=**−**14.518) but increased in the control group (MD_post-pre intervention_=3.319; *F*_1,176_=59.358, *P*<.001; *η^2^*=0.252). This effect was largely led by stress reduction (*F*_1,176_=64.679, *P*<.001; for the intervention group, MD_post-pre intervention_=**−**6.692, while for the control group, MD_post-pre intervention_=2.000). On average, participants completed 6.27 (SD 9.4) exercises and provided 9.74 (SD 18.2) mood journal logs, with a daily engagement of 4.95 (SD 6.89) minutes. However, the associations between the changes in DASS-21 scores and the numbers of exercises or mood journal logs did not reach statistical significance.

**Conclusions:**

This study primarily established the effectiveness of *Neurum* in alleviating depression, anxiety, and stress symptoms in noninstitutionalized working adults, with a satisfactory user retention rate. Despite potential health-related culture differences, *Neurum* contributed to evidence-based digital health in nonclinical settings for timely needs and general accessibility as an alternative to traditional, face-to-face, and high-cost mental health services. Future directions involving a personalized approach in online mental health services were discussed.

## Introduction

### Background

While the working population is the driving force of the global economy, common mental disorders (ie, depression and anxiety) alone have cost US $1 trillion each year due to the loss of productivity [1,2]. Pharmacotherapies, psychotherapies, or a combination of both are the primary approaches in managing the symptoms of common mental disorders [3-5]. However, suboptimal service use exists to hinder the influence of the mental health care system [6,7]. The poor mental health service use suggested an immense service gap that might not sufficiently or appropriately support the needs of individuals with mental health difficulties. Against this backdrop, it has become essential to further explore accessible, affordable, and effective alternatives to existing mental health care services for the working population.

Longitudinal studies have demonstrated that work-related stress—such as excessive workload, effort-reward imbalance, and low work social support—is associated with increased risks of depression and anxiety disorders and poor mental well-being [8-11]. Despite a large investment in mental health services and their significant effectiveness, the cost of maintaining these face-to-face interventions is high and often challenging, steering the research direction towards identifying low-cost, evidence-based strategies to optimize intervention cost-efficiency [12-18]. Worse yet, the COVID-19 pandemic has further threatened the mental health landscape of working adults by impacting individuals’ job security, chronic isolation, and uncertainty about the future, as well as adding constraints to traditional face-to-face intervention options [19]. Most of the existing interventions are limited in reaching out to those in need during a pandemic situation, increasing the risk and prevalence of mental ill health [19-22]. This limitation also applies to long-existing underuse issues of mental health services, especially in Asian countries [23,24]. For illustration, previous research in China revealed that only 24% of the participants who were diagnosed with moderate or severe mental health problems sought help from mental health professionals [25]. Reasons included issues such as health service accessibility and costs, as well as associated mental health stigmas. Therefore, mobile health (mHealth) seems a practical approach to improve the low rate of health-seeking behaviors given its advantages of being low cost, easily accessible, and stigma-free. Therefore, attention has increasingly shifted toward digital approaches as a feasible and scalable alternative.

A recent randomized controlled trial (RCT) showed that web-based mindfulness and cognitive behavioral training were efficacious in alleviating psychological distress among college students and young working adults [26]. Several meta-analyses suggested that web-, application-, and/or computer-based interventions were effective in alleviating depression, anxiety, and stress, though with undeniably concerning attrition rates ranging widely from 43% to 99% [27-31]. Taken together, these findings pointed out the effectiveness of internet-based psychological interventions while urging further refinement of their user retention especially for long-term benefits. In addition to internet-based interventions, mHealth platforms that adopt mobile and wireless devices to improve health care also appear promising [32]. Not only has mHealth long been used in physical health care, such as for diabetes management, infectious disease management, and immunization management, increasing application of mHealth to mental health also has emerged, ranging from alcohol and substance use assessment to low-intensity interventions for mental disorders [33-38]. Despite the enlightening trend, less than 5% of the mHealth platforms underwent peer-reviewed research, while the majority of these were conducted in western countries [39,40]. Peer-reviewed, demographically diverse research awaits attention.

Advancements, availability, and affordability of mobile technology have given rise to ecological momentary assessment (EMA), which allows individuals to report experiences and behaviors in everyday lives and in naturalistic settings [41,42]. The rationale for using EMA rests upon 3 major advantages including reducing retrospective recall, improving generalizability and ecological validity, and enabling the exploration of temporal relationships among variables. Similarly, ecological momentary intervention (EMI) refers to the delivery of intervention while individuals are naturally engaged in their everyday lives [43,44]. Emerging evidence suggested the effectiveness of EMI in alleviating anxiety and stress and treating a variety of health behaviors and psychological symptoms [44,45]. However, RCTs of EMA and EMI in mental health promotion remained scarce [45]—and their application in workplace mental health services is even more limited. This is noteworthy given that both approaches could offer unique benefits to working adults, as they deliver mental health care services free from temporal and spatial constraints. Therefore, this gap underscores the need for rigorous investigation of EMA- and EMI-based mHealth tools, particularly in workplace populations and non-Western contexts.

### Objectives

To address this gap, this preregistered RCT was designed to evaluate the effectiveness of a smartphone-based intervention (ie, the *Neurum* app; Neurum Limited) that leverages both EMA and EMI in reducing workplace stress [46]. Study hypotheses and corresponding analytical schemes were preregistered in September 2020 (registration reference: osf.io/z829u) before the data collection was finished. No statistical analysis was done before the preregistration or the completion of data collection. Specifically, it was hypothesized that participants who received intervention through the *Neurum* app would display better workplace stress management and mental health improvement as reflected by the reduction in depression, anxiety, and/or stress symptoms. The design, conduct, and reporting of this study followed the CONSORT-EHEALTH (Consolidated Standards of Reporting Trials of Electronic and Mobile Health Applications and Online Telehealth) guidelines (Checklist 1).

## Methods

### Participants

This study was a 2 (between: intervention vs waitlist control groups) × 2 (within: premeasures vs postmeasures) mixed factorial design. A priori power analysis using *F* test family (repeated measures, between factors) in G*Power was conducted [47]. Assuming a small effect size (ie, *f*=0.25) and a high correlation between premeasures and postmeasures of the outcome variable (ie, *r*=.85), with an α level of .05, a total number of 196 participants was needed to achieve a power of 0.80. Assuming an attrition rate of 25%, a sample of 262 participants was determined prior to data collection.

Participant recruitment started in September 2019, Hong Kong SAR. Advertisements were posted on the company websites and populated through themed webinars and offline workshops to reach local working adults. However, due to local social unrest and the COVID-19 pandemic, few signed up within the planned 1-month recruitment period. Therefore, under all authors’ and the funding agency’s approval, the recruitment process was prolonged until the deemed sample size would be achieved. The recruitment process lasted for over a year and was finally terminated by the end of October 2020, when nearly no more participants signed up within 30 days. To maximize the sample size, those who signed up and passed the screening immediately underwent the intervention process. However, no data analysis was conducted before the end of the study. In total, 201 working adults were recruited from a selected list of local companies, who were first directed to an online survey with screening questions, in which they reported their demographic and mental health information. A research assistant who remained unaware of the study design was in charge of the eligibility screening. Specifically, participants were eligible if they (1) were aged 18 years or older at sample recruitment, (2) had access to the internet and a smartphone, and (3) were not diagnosed with major mental disorders. Exclusion criteria included (1) mental illness diagnosis history, (2) current reception of professional mental health services, or (3) current use of psychotropic medication.

### Ethical Considerations

Ethical approval was obtained from the Subcommittee on Research Ethics and Safety of the Research Committee of Lingnan University (reference: EC047/2021). All procedures contributing to this work complied with the ethical standards of the relevant national and institutional committees on human study. Informed consent was obtained via electronic signatures indicating the awareness of the study procedure, potential risks and benefits, data confidentiality, and the right to quit the study at any time. Data were encrypted and stored in a financial-sector security level server and deidentified before statistical analysis. No incentives or compensations were provided.

### Procedure

All procedures were delivered via *Neurum*. Included participants first completed the preintervention assessment, and then they were randomly allocated at a 1:1 ratio to the two groups via a computer algorithm of simple randomization. Specifically, a numerical value between 0 and 1 was assigned to each participant. Those who received a value smaller than 0.5 were assigned to the control group; otherwise, participants were assigned to the intervention group. Double blindness was achieved such that a research assistant who remained unaware of the study design was in charge of the participant assignment, while participants received a unique study ID in a singular format that revealed no group information, and therefore, remained unaware of the grouping. Then, participants in the intervention group received EMA and EMI via *Neurum* for 4 weeks, while those in the waitlist control group were offered the intervention after the RCT was completed. Finally, all participants completed a postintervention assessment with debrief at the end of the trial (ie, after the designated 4-week intervention [or control] duration).

### Intervention

*Neurum* is a smartphone app that comprises self-managed mental health modules designed in various combinations of (1) featured techniques, namely cognitive behavioral therapy, mindfulness exercises, and self-regulation skills with (2) various session formats including informational (ie, psychoeducation), evaluative (ie, assessment), and applicable (ie, exercise), aiming at (3) tangible goals in alignment with the biopsychosocial model, namely the improvement and maintenance of biological, psychological, and social health. See Table 1 for a summary of the different components and the rationale.

**Table 1.** *Neurum* intervention framework and module examples.

Example module	Featured technique	Format		
		Psychoeducation	Assessment	Exercise
Biological				✓
Acupressure	Self-regulation			✓
Sleep quality	Mindfulness	✓		✓
Psychological				
Focus timer	Self-regulation			✓
Managing stress	Cognitive-behavioral therapy	✓	✓	✓
Sustaining happiness	Mindfulness	✓		✓
Social				
Workplace boundaries	Self-regulation	✓	✓	✓

*Neurum* is designed with an instant messenger–like user interface where users interact with the virtual app assistant in a conversation format. The palatability of the dialogues was ensured by using layman and local terms in either English or Chinese. The app content was developed with standard app development iterations that involved digitizing of modules based on previously validated mental health education and behavioral changing paradigms, and then user testing with an A/B test method to compare the performance of 2 versions of content. This study describes the foundation of the app, which matches users to an EMI based on information from their EMA via a combination of supervised learning models, but future iterations of *Neurum* involved personalization of EMI modules based on nuances from EMA, session feedback, and other baseline user data via a more complex deep learning algorithm. As part of the safeguarding protocol, a referral mechanism is established, such that users who score past a certain threshold in their levels of psychological distress are automatically referred to crisis management protocol, which involves the algorithm providing the user with immediate support via distress reduction modules, crisis hotlines, and face-to-face care delivered by a licensed, on-board clinical psychologist. No adverse events were reported during the study.

**Figure 1.**
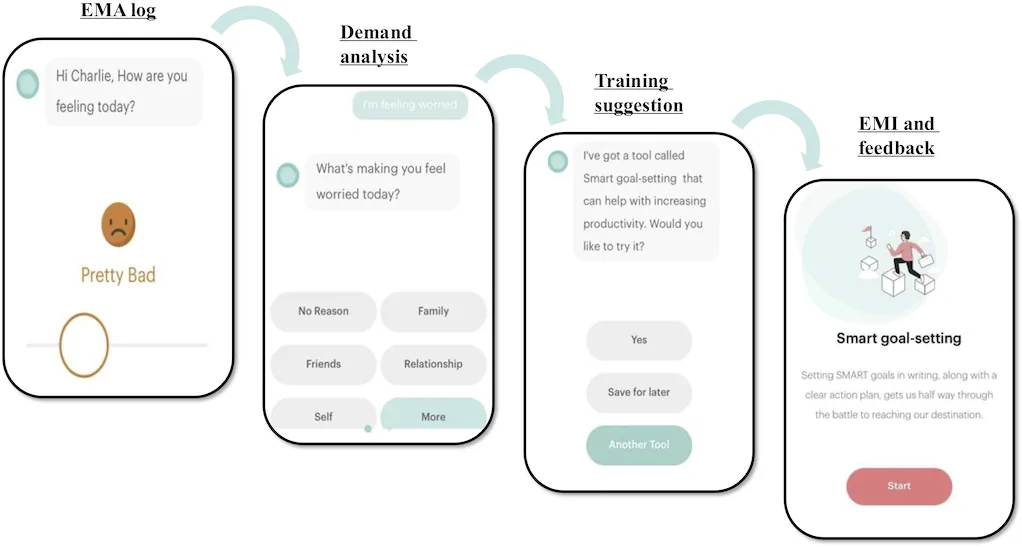
Illustration of ecological momentary assessment (EMA) and ecological momentary intervention (EMI) workflow on the Neurum (Neurum Limited) app.

EMA is delivered such that users are invited to complete a mood journal on *Neurum* to log their instant emotions, thoughts, and behaviors relevant to workplace stress as well as other psychological distress whenever they feel in need. Users evaluate their general feelings along a negative-to-positive continuum, specify a specific experience they would like to work on, and then identify one or more associated stressors. EMI is delivered such that, upon EMA logs, *Neurum* would make a recommendation to relevant, evidence-based intervention modules based on an immediate matching of the current demand and available training tools using a self-developed, built-in supervised machine learning algorithm. User feedback is further collected after each training session, which also informs the algorithm. A daily summary of the reported mood experiences and taken exercises was provided for self-monitoring of workplace stress and management performance. See Figure 1 for the overview of *Neurum*’s EMA or EMI workflow.

### Measures

#### Depression, Anxiety, and Stress

Key outcomes, namely the intervention effectiveness, were evaluated by comparing the prescores and postscores of the Depression, Anxiety, and Stress Scale-21 items (DASS-21) [48]. Participants rated the extent to which they experienced depression, anxiety, and stress symptoms (7 items for each dimension; eg, “I was worried about situations in which I might panic”) on a 4-point scale (0=“Did not apply to me at all” to 3=“Applied to me very much or most of the time”). Sum scores were computed for the 3 subdimensions as well as the total scores such that higher scores reflected more severe symptoms. The adopted Chinese version of the DASS-21 exhibited satisfactory psychometric properties (Cronbach α ≥0.80) [49]. Cronbach α in this study was 0.87.

#### Mood Journal

Participants in the intervention group were invited to report, every time they logged in, on (1) how they were feeling in general; (2) whether they were feeling any specific emotion, such as happy or disappointed; and if yes, (3) the source of emotion such as family, work, or friends.

#### User Activity

User activity was measured by the number of mood journals completed and exercise sessions taken, as well as daily user engagement (in min).

#### Other Measures

At baseline, participants provided demographic information including sex, age, and marital status.

### Analytical Scheme

To examine the intervention effect, pre– and post–DASS-21 scores of the intervention and control groups were submitted to a 2 (between: intervention vs control groups) × 2 (within: premeasures vs postmeasures) mixed ANOVA. Additional analyses included categorical demographic characteristics (namely sex, education, and marital status; Table 2) as control variables. A supplementary set of mixed ANOVA was conducted to compare intensity level changes for each symptom. Finally, 2 multiple linear regressions with a number of mood journals and exercise sessions completed were conducted to explore any dose-response effect.

**Table 2.** Demographic characteristics and baseline Depression, Anxiety, and Stress Scale-21 items (DASS-21) scores for each group.

Characteristics	Control group (n=97)	Intervention group (n=81)
Age (y), mean (SD)	34.65 (7.67)	36.43 (12.84)
Sex, n (%)		
Female	51 (53)	42 (52)
Male	46 (47)	39 (48)
Education, n (%)		
High school	5 (5)	8 (10)
Bachelor’s degree	64 (66)	48 (59)
Master’s degree	0 (0)	18 (22)
PhD or above	6 (6)	3 (4)
Prefer not to tell	22 (23)	4 (5)
Marital status, n (%)		
Single	54 (56)	40 (49)
Married	33 (34)	38 (47)
Divorced	7 (7)	2 (2)
Separated	3 (3)	0 (0)
Prefer not to tell	0 (0)	1 (1)

## Results

### Sample Characteristics

**Figure 2.**
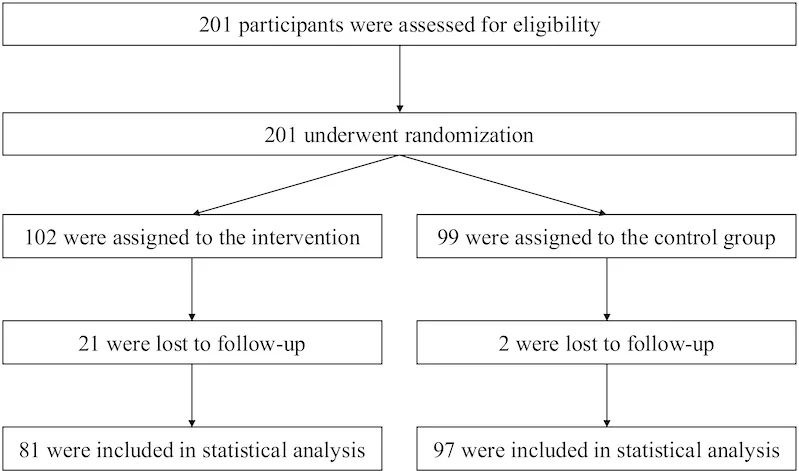
Participant flow diagram.

Figure 2 shows the participant flow diagram. Demographic characteristics are summarized in Table 2. The majority of study participants were females, had bachelor’s degrees, and were single in terms of marital status. Though the overall attrition rate was low (23/201, 11.4%), there were significantly more participants dropped from the intervention group (n=21) than from the control group (n=2; *χ*^2^_1_=18.259; *P*<.001), resulting in 81 participants in the intervention group and 97 in the control group. Considering the intensive EMA or EMI procedures, the differential attrition rates between the 2 groups were not surprising. More importantly, the final sample consisted of 178 working adults and was still sufficient in statistical power.

### User Activity

On average, participants in the intervention group provided 9.74 (SD 18.2; maximum=130) mood journals and completed 6.27 (SD 9.4; maximum=51) exercises, while neither was correlated to preintervention DASS-21 scores (|*r*|s ≤.156; *P*s≥.16), and neither was predicted by sex (*t*_79_s≤0.744; *P*s≥.46), education level (*F*_3, 73_s≤1.947; *P*s≥.13), or marital status (*F*_2, 77_s≤2.002, *P*s≥.14). Daily user engagement on average was 4.95 (SD 6.89; maximum=30) minutes, which was positively correlated with preintervention stress scores (*r*=0.233; *P*=.002) and total DASS-21 scores (*r*=0.175; *P*=.02), but was not predicted by sex (*t*_78_=0.791; *P*=.22), educational level (*F*_3, 73_=1.595; *P*=.20), or marital status (*F*_2, 76_=0.100; *P*=.91).

### Intervention Effects

**Figure 3.**
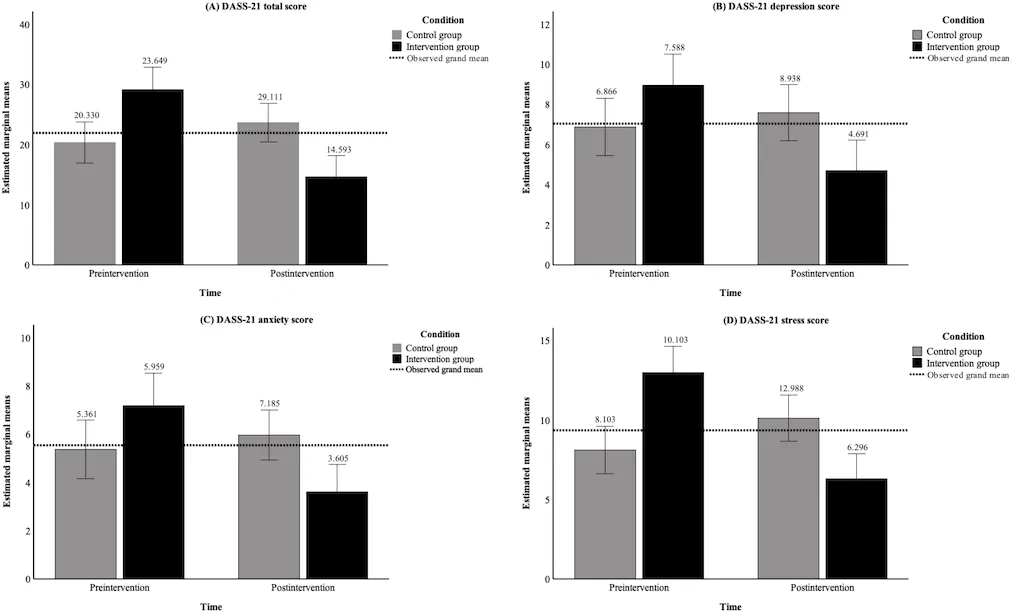
Preintervention and postintervention Depression, Anxiety, and Stress Scale-21 items (DASS-21) results including (A) total score and (B) depression, (C) anxiety, and (D) stress subdimensional scores between intervention and control groups. Error bars represent 95% CIs.

Preintervention and postintervention DASS-21 scores, as well as the mean differences, are presented in Table 3. Mixed ANOVAs were applied to compare the pre– and post–DASS-21 total and subscale scores between groups (Table 4). For the total score, equal variance between groups was satisfied (Levene statistics ≤2.127; *P*≥.15). Results revealed that there was no significant difference between the intervention (mean difference [MD]=21.990) and the control (MD=21.852) groups; but as expected, there was a significant postintervention decrease (MD_preintervention_=24.721 and MD_postintervention_=19.121). More importantly, the above effects were quantified by a significant, large-effect size interaction between time and group (Figure 3A) such that the DASS-21 total scores decreased for the intervention group (MD_preintervention_=29.111 and MD_postintervention_=14.593) but increased for the control group (MD_preintervention_=20.330 and MD_postintervention_=23.649). Similar patterns (Figure 3B-D) were found for the three symptoms such that average scores notably decreased for the intervention group but slightly increased for the control group (*F*_1, 176_s≥22.689, *P*s<.001), with the strongest effect observed in the stress symptom.

**Table 3.** Preintervention and postintervention scores and mean differences between groups (n_control_=97 and n_intervention_=81).

Variable	Preintervention, mean (SD)		Postintervention, mean (SD)		Mean difference^a^	
	Control	Intervention	Control	Intervention	Control	Intervention
Depression	6.866 (6.843)	8.938 (7.417)	7.588 (7.071)	4.691 (7.015)	0.722	−4.247
Anxiety	5.361 (6.289)	7.185 (5.812)	5.959 (5.752)	3.605 (4.398)	0.598	−3.580
Stress	8.103 (7.248)	12.988 (7.76)	10.103 (7.796)	6.296 (6.520)	2.000	−6.692
Total	20.33 (16.283)	29.111 (17.975)	23.649 (16.857)	14.593 (15.174)	3.319	−14.518

a Mean differences are computed as postintervention minus preintervention scores.

**Table 4.** Mixed ANOVAs of preintervention and postintervention Depression, Anxiety, and Stress Scale-21 items (DASS-21) scores between groups (n_control_=97 and n_intervention_=81).

Outcome	*F* test (*df*)	*P* value	η^2^
Total			
Between			
Group	0.004 (1, 176)	.95	0.000
Within			
Time	23.396 (1, 176)	<.001	0.117
Time × group	59.358 (1, 176)	<.001	0.252
Depression			
Between			
Group	0.197 (1, 176)	.66	0.001
Within			
Time	11.422 (1, 176)	.001	0.061
Time × group	22.689 (1, 176)	<.001	0.114
Anxiety			
Between			
Group	0.122 (1, 176)	.73	0.001
Within			
Time	15.182 (1, 176)	<.001	0.079
Time × group	29.800 (1, 176)	<.001	0.145
Stress			
Between			
Group	0.310 (1, 176)	.58	0.002
Within			
Time	18.844 (1, 176)	<.001	0.097
Time × group	64.679 (1, 176)	<.001	0.269

### Supplementary Analyses

A set of supplementary models found similar patterns controlling for sex, education, and marital status (for condition effects, *F*_1, 146_s≤1.601, *P*s≥.21, *η^2^*≤0.011; for time effects, *F*_1, 146_s≥2.933, *P*s≤.09, *η^2^*≥0.020; for interaction effects *F*_1, 146_s≥19.286, *P*s≤.001, *η^2^*≥0.117), suggesting that the above intervention effects held after controlling for these demographic covariates. Another set of supplementary models (Table 5) found similar patterns using DASS-21 symptom intensity levels as the outcome variables. Change(s) of these intensity levels suggested that the control group ascended while the intervention group descended in symptom severity (*χ*^2^_2_s≥ 8.605; *Ps*≤.02). Finally, and surprisingly, a final set of supplementary models (Table 6) using multiple linear regressions revealed that none of the exercise session number, mood journal number, or average daily engagement predicted the postintervention DASS-21 scores.

**Table 5.** Intensity level changes between groups (n_control_=97 and n_intervention_=81)^a^.

	Intensity level change			Chi-square (*df*)	*P* value
	Worsen, n (%)	No change, n (%)	Improved, n (%)		
Depression					
Control	17 (18)	64 (66)	16 (16)	8.605 (2)	.02
Intervention	9 (11)	44 (54)	28 (35)		
Anxiety					
Control	21 (22)	67 (69)	9 (9)	27.581 (2)	<.001
Intervention	5 (6)	43 (53)	33 (41)		
Stress					
Control	19 (20)	72 (74)	6 (6)	23.404 (2)	<.001
Intervention	4 (5)	52 (64)	25 (31)		

a Cell values in each intensity change category are counts of participants.

**Table 6.** User activity and postintervention Depression, Anxiety, and Stress Scale-21 items (DASS-21) scores for the intervention group (n=81).

	B	SE	*P* value	95% CI
Total score^a^				
Model 1				
Baseline level	0.220	0.092	.02	0.037 to 0.403
Model 2				
Baseline level	0.224	0.093	.02	0.038 to 0.410
Exercise session	−0.073	0.194	.71	*−*0.459 to 0.313
Mood journal	0.120	0.099	.23	−0.077 to 0.318
Daily engagement	0.240	0.253	.35	−0.263 to 0.744
Depression^b^				
Model 1				
Baseline level	0.286	0.102	.006	0.083 to 0.489
Model 2				
Baseline level	0.296	0.105	.006	0.086 to 0.505
Exercise session	0.001	0.088	.99	−0.175 to 0.177
Mood journal	0.027	0.046	.59	−0.064 to 0.119
Daily engagement	0.029	0.117	.80	−0.204 to 0.262
Anxiety^c^				
Model 1				
Baseline level	0.241	0.081	.004	0.080 to 0.403
Model 2				
Baseline level	0.228	0.083	.008	0.062 to 0.394
Exercise session	−0.033	0.056	.55	−0.144 to 0.078
Mood journal	0.018	0.029	.52	−0.038 to 0.075
Daily engagement	0.086	0.073	.25	−0.060 to 0.232
Stress^d^				
Model 1				
Baseline level	0.201	0.092	.03	0.018 to 0.385
Model 2				
Baseline level	0.200	0.092	.03	0.018 to 0.383
Exercise session	−0.035	0.082	.67	−0.199 to 0.128
Mood journal	0.077	0.042	.08	−0.007 to 0.160
Daily engagement	0.129	0.108	.23	−0.085 to 0.343

a For model 1, adjusted *R*2=0.068, *F*_1,78_=5.731, *P*=.02; and for model 2, adjusted *R*2=0.097, *F*_1,72_=0.777, *P*=.51.

b For model 1, adjusted *R*2=0.080, *F*_1,78_=7.855, *P*=.006; and for model 2, adjusted *R*2=0.049, *F*_1,72_=0.162, *P*=.92.

c For model 1, adjusted *R*2=0.091, *F*_1,78_=8.866, *P*=.004; and for model 2, adjusted *R*2=0.076, *F*_1,72_=0.601, *P*=.62.

d For model 1, adjusted *R*2=0.045, *F*_1,78_=4.762, *P*=.03; and for model 2, adjusted *R*2=0.067, *F*_1,72_=1.593, *P*=.20.

## Discussion

### Overview

This RCT examined the effectiveness of a smartphone-based EMA or EMI mental health care services delivered by the *Neurum* app in mitigating workplace stress and improving mental health for working adults in Hong Kong SAR, China. Consistent with the hypotheses, results demonstrated that the approach was effective in reducing psychological distress after 4 weeks of intervention, suggesting that the EMA or EMI approach via the *Neurum* app could support working adults’ stress management in real time and in naturalistic settings.

### Principal Findings

This study demonstrated the effectiveness of integrating EMA or EMI techniques with supervised machine learning algorithms in digital mental health care for a nonclinical population. The findings were broadly compatible with previous studies supporting the effectiveness of mHealth tools in reducing general anxiety, various stress, and depression symptoms [45,50]. Importantly, the effectiveness of *Neurum* was supported not only by the continuous reduction of psychological symptoms but also by the shifts in symptom intensity levels, suggesting potential use that extends from non–clinical contexts into early-stage clinical applications. One well-known advantage of the ecological momentary approach is its ability to minimize retrospective bias and enhance event trigger monitoring. Previous research has highlighted the promise of such digital phenotyping for routine clinical assessment, particularly for early onset or relapse [51]. Our observed changes in categorical symptom intensity levels further supported this clinical relevance, indicating that symptom improvement was substantial enough to cross the established cutoff thresholds. Because categorical severity shifts could provide a more interpretable indicator of treatment effectiveness than numerical score changes alone, these findings underscore the potential of EMA or EMI-based mHealth tools for early identification, timely intervention, and preventative health care in mental health. Additionally, previous meta-analyses research revealed that multicomponent intervention was tentatively more effective than single-component ones (eg, with cognitive behavioral therapy alone), and that patient preferences for specific intervention components played a pivotal role in treatment outcomes [52,53]. In response to these challenges, the intervention delivered in *Neurum* integrated multiple modules in various delivery formats for actionable outcomes, enabling participants to decide whether and which exercises to engage in upon each log-in. This inclusive design undoubtedly promoted user retention and engagement and maximized the alignment between individual needs and intervention content, a pattern supported by our user activity data. Taken together, these novel findings highlighted the importance of developing scalable, personalized mHealth tools as accessible, affordable, and flexible alternatives to face-to-face mental health care services.

There were several important issues in the interpretation of results. First of all, more participants dropped out of the intervention group than from the control group. It is noteworthy that the overall dropout did not exceed the predetermined attrition rate for power analysis. It is also noteworthy that the current attrition rate (ie, 11.4%) was smaller than those reported in previous studies (eg, about 26% for cognitive behavioral therapy and about 19% for mindfulness-based traditional interventions, or widely ranging from 43% to 99% for general digital health interventions [27-31,54-56]). Despite this, authors speculated the presence of some exposure effects particularly for the intervention group. Specifically, EMA or EMI required participants to review their mood swings on a daily basis, to exert efforts in managing especially those negative daily experiences, and to review their own performances on the taken exercises. Compared to those assigned to the control groups, these tasks might make the mental health issues more evident than they used to be (namely, before participating in this study) for the intervention group, possibly resulting in a withdrawal from treatment effect frequently observed in traditional psychotherapies [57]. Regrettably, the current design did not include relevant measures to test this speculation. However, to improve its future retention, the intervention could benefit from including methods to cope with the aforementioned exposure effect in the onboarding stage. Meanwhile, a personalized approach that maps personal needs with module recommendations can also improve user retention and performance. In fact, future iterations of *Neurum* have involved personalization of EMI modules based on nuances from EMA, session feedback, and other baseline user data via a more complex deep learning algorithm. Second, the results somewhat failed to support the association between the number of completed exercises or mood journals with DASS-21 score changes. In other words, the underlying dose-response effect remained unclear. One possible reason was that participants were only *encouraged* but not forced to report their daily mood journals or to take exercises. Granted that it strategically maximized participants’ acceptance and adherence [58], it undeniably resulted in the limitation of a relatively low daily response rate, and subsequently, the lack of statistical power to reveal the targeted dose-response effect, if any. Therefore, future studies might consider assigning a compulsory number of daily responses to unveil potential underlying mechanisms. Despite this, the current intervention effects could be a result of the improved awareness of one’s own mental health status and consequently improved proactive mental health management effort, or an effect generalized from in-app exercises to realistic life scenarios.

This study also contributes to the relatively nascent field of mHealth research, typically the application of EMA or EMI, in Asian countries [40]. Though with a rapid growth and a wide application, empirical supports of EMA or EMI for mental health were mostly from the United States and European countries (eg, Germany and the Netherlands), with the only exception being a study involving Chinese participants with mild-moderate depression [59-61]. However, western-based mHealth apps were not necessarily applicable to eastern ones [62]. The common mHealth apps in Asian contexts have remained in psychoeducation, counseling services, and self-assessment tools [40,63], while this study expanded them by leveraging the more intense EMA or EMI approach. Furthermore, *Neurum* takes health equity and cultural adaptivity into consideration by offering multiple language versions (viz, both traditional and simplified Chinese, English, Filipino, and Hindi) to serve local people with different cultural backgrounds. Though ethnicity was not collected in this investigation, the recruitment process was indeed carried out in international enterprises in Hong Kong, SAR. Finally, this study expanded the use of EMA or EMI beyond clinical settings to community populations, which broadened the spectrum of the recipients of such digital health care services. While some digital health tools can be used by patient populations to clinically reduce their depression and/or anxiety, this must be differentiated from digital health tools that are meant to reduce these symptoms in a non–clinical population. The demonstrated effectiveness of *Neurum* is similar to other well-being and mindfulness apps, such as *Calm* and *Headspace* [64-66]. Furthermore, the function of these digital health platforms and the reduction in mental health symptoms are similar to those of other non–clinical interventions like yoga [67]. The wellness technology industry has been growing exponentially with app-based options that are not designed to replace clinical treatment altogether, but to offer complementary early intervention tools, or as alternative options for those that struggle to access in-person care. Relatedly, *Neurum* essentially represents a shift from disease-centered to health-centered, proactive mental health approach. EMA or EMI, combined with diverse delivery formats (namely, informational, evaluative, and practical), comprehensively supports a proactive mental health management system by addressing foundational mental health literacy, enabling timely monitoring, fostering essential skills, and promoting sustained actions. Such a shift of medical models for mental health corroborates the notion of prevention over treatment, as globally advocated by many governmental and regional health policies [68-71].

### Limitations

This study has several limitations. First, despite the authors’ effort to recruit participants and maintain their engagement during the local social unrest and the COVID-19 pandemic, the final sample size was still slightly smaller than expected. However, the effect size of mixed ANOVA was large, supporting the effectiveness of the intervention. Second, even though *Neurum* was designed with multiple languages, ethnicity information was not collected, which precluded the possibility of examining cultural differences, if any. Factors such as mental health beliefs, perceptions, and literacy should merit future research attention to improve the generalizability of *Neurum* in other cultures [72,73]. Third, while the attrition rate of the current study was low, local RCTs on web-based and application-based mental health programs suggested that personalized feedback might mitigate the high attrition rates typically in internet-based programs [26,74]. Indeed, several lines of evidence demonstrated the benefits of personalized intervention including better intervention outcomes, stronger subject adherence, and more persuasive feedback, in both physical and mental health domains [62,75-78]. As such, *Neurum* could be further advanced by incorporating the personalized intervention approach based on computational algorithms of the EMA or EMI data. Finally, the results of this study did not reveal a dose-response relationship between the reductions in psychological symptoms and the number of EMI sessions completed. A requirement of minimum sessions in the future can help examine the potential dose-response effect and better understand the intervention’s effectiveness.

### Conclusions

In sum, this RCT supported that *Neurum*, a new mHealth intervention that coupled the ecological momentary approach with supervised machine learning algorithms, could effectively reduce workplace stress and psychological distress in working adults. Beyond establishing its efficacy, the findings also highlight the potential of day-to-day, context-sensitive data to deepen the understanding of how EMA or EMI functions within digital psychological interventions. By introducing inclusive design and diverse user groups, this work further underscores the importance of equity and cultural relevance in the next generation of digital mental health care. Together, these insights highlight the promise of adaptive, data-driven mHealth tools in expanding the access to effective psychological support across different populations and in real-time and real-world settings.

## Supplementary material

10.2196/72435 Checklist 1 CONSORT-EHEALTH checklist.

## Abbreviations

CONSORT-EHEALTH: Consolidated Standards of Reporting Trials of Electronic and Mobile Health Applications and Online Telehealth
DASS-21: Depression, Anxiety, and Stress Scale-21 items
EMA: ecological momentary assessment
EMI: ecological momentary intervention
MD: mean difference
mHealth: mobile health
RCT: randomized controlled trial
