# Supplementary material for: A Smartphone-Based Ecological Momentary Intervention for Workplace Mental Health: Randomized Controlled Trial
Source: JMIR Mhealth Uhealth. 2026 Sep 8;14:e72435. doi: 10.2196/72435 (PMC13552837; doi:10.2196/72435)
Supplement: Supplementary file 1 [file mhealth-v14-e72435-s001.pdf]

CONSORT-EHEALTH Checklist V1.6.2 Report

Manuscript Number 72435

(based on CONSORT-EHEALTH V1.6), available at [http://tinyurl.com/consort-ehealth-v1-6].

Date completed
3/19/2025 10:20:18
by
Sisi Li

TITLE

1a-i) Identify the mode of delivery in the title

1a-ii) Non-web-based components or important co-interventions in title
Yes, "a smartphone-based intervention" which pointed out the intervention was delivered via smartphone.

1a-iii) Primary condition or target group in the title
Not applicable, as the intervention was delivered entirely online.

ABSTRACT

1b-i) Key features/functionalities/components of the intervention and comparator in the METHODS section of the ABSTRACT

1b-ii) Level of human involvement in the METHODS section of the ABSTRACT
Yes, "a 4-week smartphone-based intervention which incorporated ecological momentary assessment/intervention with psychoeducation, cognitive-behavioral therapy, and mindfulness exercises".

1b-iii) Open vs. closed, web-based (self-assessment) vs. face-to-face assessments in the METHODS section of the ABSTRACT
Yes, this intervention was delivered mainly automated except for the referral mechanism where a licensed, on-board clinical psychologist would provide supplementary, face-to-face crisis management when necessary. Though it was included in the abstract due to word limits, this information was reported in the manuscript (see lines 186-190).

1b-iv) RESULTS section in abstract must contain use data
Yes. This study was a closed usergroup trial as participants were recruited from a closed online user group, "A total of 201 working adults were recruited...". And outcomes were measured via self-assessment. "Key outcome was measured by the Depression, Anxiety, and Stress Scale (DASS-21; Cronbach's α = 0.87)."

1b-v) CONCLUSIONS/DISCUSSION in abstract for negative trials
Yes. For attrition rate, "The intervention group consisted of 102 participants while the control group consisted of 99 participants. More participants dropped from the intervention (n = 21) group than from the control group (n = 2; $\chi^2(1) = 18.259$, $P < .001$)." For use over time, " On average, participants completed 6.27 exercises and provided 9.74 mood journals."

INTRODUCTION

2a-i) Problem and the type of system/solution

2a-ii) Scientific background, rationale: What is known about the (type of) system
Yes, "This study was a pre-registered randomized controlled trial to evaluate the effectiveness of a smartphone-based intervention (i.e., the Neurum app) which leverages both EMA and EMI in reducing workplace stress". Additionally, this study was a stand-alone intervention as a complement for universal, traditional, face-to-face mental health services.

METHODS

3a) CONSORT: Description of trial design (such as parallel, factorial) including allocation ratio

3b) CONSORT: Important changes to methods after trial commencement (such as eligibility criteria), with reasons
Yes, "Specifically, it was hypothesized that participants who received intervention through Neurum would display better workplace stress management and mental health improvement as reflected by the changes in the DASS-21 scores.".

3b-i) Bug fixes, Downtimes, Content Changes

4a) CONSORT: Eligibility criteria for participants
Yes, "This study was a 2 (between: intervention vs. waitlist control groups) × 2 (within: pre- vs. post- measures) mixed factorial design". Additionally, participants were then randomly allocated at a 1:1 ration to the two groups. This information was not made explicit in our submitted version and we are happy to add it in later revision process.

4a-i) Computer / Internet literacy

4a-ii) Open vs. closed, web-based vs. face-to-face assessments:
Yes, "(2) had access to the Internet and a smartphone"

4a-iii) Information giving during recruitment
Yes. This study was a closed usergroup trial as participants were recruited from a closed online user group, "A total of 201 working adults were recruited...". And outcomes were measured via self-assessment. "Key outcome was measured by the Depression, Anxiety, and Stress Scale (DASS-21; Cronbach's α = 0.87)."

4b) CONSORT: Settings and locations where the data were collected
No. We would like to clarify here that participants were provided with a fixed list of exercises without bug fixes or content changes during the study.

4b-i) Report if outcomes were (self-)assessed through online questionnaires

4b-ii) Report how institutional affiliations are displayed
Yes, "Key outcome was measured by the Depression, Anxiety, and Stress Scale (DASS-21; Cronbach's α = 0.87)." But we would like to make it more explicitly in the next round of revision by mentioning that the outcome was assessed via the Neurum.

5) CONSORT: Describe the interventions for each group with sufficient details to allow replication, including how and when they were actually administered

5-i) Mention names, credential, affiliations of the developers, sponsors, and owners

5-ii) Describe the history/development process
Yes, the developer name was included in the manuscript.

5-iii) Revisions and updating
No, history/development of the application was not included but we would like to report in it is highly recommended during the revision.

5-iv) Quality assurance methods
No. We would like to clarify here that participants were provided with a fixed list of exercises without app update during the study.

5-v) Ensure replicability by publishing the source code, and/or providing screenshots/screen-capture video, and/or providing flowcharts of the algorithms used
Yes, procedues and protocols are reported in the manuscript.

5-vi) Digital preservation
Yes, please refer to Figure 2 for the app flowchart. The algorithms cannot be reased due to copyright issues. However, more elaboration would be provided for review process if necessary.

5-vii) Access
Yes, as in reference #46, "Neurum Limited. (2023). Neurum [Mobile app]. App store. https://www.neurumhealth.com/. doi: N.A. PMID: N.A.".

5-viii) Mode of delivery, features/functionalities/components of the intervention and comparator, and the theoretical framework

Yes, as quoted here, "while participants received a unique study ID in a singular format that revealed no group information, and therefore, remained unaware of the grouping. All participants completed a baseline survey including socio-demographic information and mental health assessment. Then, participants in the intervention group received EMA coupled with EMI via Neurum for four weeks, while those in the waitlist control group did not receive any intervention until the end of the four-week intervention duration. ". Additionally, authors would like to clarify that participants accessed this App using this unique study ID on their smartphone, no fee was charged. If reviewers would like to request a demo account, pelase let the authors know.		
**5-ix) Describe use parameters**		
Yes, as quoted here, "......Neurum is a smartphone application which comprises modules about psychoeducation, cognitive-behavioral therapy, and mindfulness exercises, validated by behavioral health scientists together with clinical psychologists and deemed appropriate for reducing workplace stress. Neurum is designed with an instant-messenger-like user interface where users interact with the virtual App assistant in a conversation format. Palatability of the dialogues was ensured by using layman and local terms in either English or Chinese. ...... Ecological momentary assessment was delivered such that users could log their instant emotions, thoughts, and behaviors relevant to workplace stress as well as other psychological distress whenever they felt in need. Participants would evaluate their general feelings along a negative-to-positive continuum, specify a specific emotion they would like to work on, and then identify one or more associated stressors. Daily summaries were provided for self-monitoring of stress experiences, along with psycho-education sessions themed on relevant stress and coping skills. Upon EMA logs, Neurum would make a recommendation to the relevant intervention module based on an immediate matching of the current demand and available training tools using a self-developed, built-in machine learning algorithm. User feedback was collected after each training session, which also informs the machine learning algorithm. ......", Please also see Figure 2 for an overview of the EMA and EMI workflow on this app.		
**5-x) Clarify the level of human involvement**		
Yes, the intervention was used ad libitum and whenvener participants in the intervention group felt a need to do so, as quoted here, " Ecological momentary assessment was delivered such that users could log their instant emotions, thoughts, and behaviors relevant to workplace stress as well as other psychological distress whenever they felt in need. Participants would evaluate their general feelings along a negative-to-positive continuum, specify a specific emotion they would like to work on, and then identify one or more associated stressors. Daily summaries were provided for self-monitoring of stress experiences, along with psycho-education sessions themed on relevant stress and coping skills. Upon EMA logs, Neurum would make a recommendation to the relevant intervention module based on an immediate matching of the current demand and available training tools using a self-developed, built-in machine learning algorithm. User feedback was collected after each training session, which also informs the machine learning algorithm. "		
**5-xi) Report any prompts/reminders used**		
Yes, as quoted here, "As part of the protocol, a referral mechanism was established, such that users who score past a certain threshold in their levels of distress are automatically referred to our crisis management protocol, which involves the algorithm providing the user with immediate support via distress reduction modules, crisis hotlines, as well as face to face care delivered by a licensed, on-board clinical psychologist. No adverse events were reported during the study.".		
**5-xii) Describe any co-interventions (incl. training/support)**		
Yes, Yes, the intervention was used ad libitum and whenvener participants in the intervention group felt a need to do so. Please also see subitem 5-ix.		
**6a) CONSORT: Completely defined pre-specified primary and secondary outcome measures, including how and when they were assessed**		
Yes, "Participants were eligible if they (1) aged 18 years or above at sample recruitment; (2) had access to the Internet and a smartphone; (3) were not diagnosed with major mental health problems. Exclusion criteria included (1) mental illness diagnosis history; (2) current reception of professional mental health services; (3) current use of psychotropic medication. Recruitment advertisements were posted on local social media, company websites, and were populated through themed webinars and offline workshops. "		
**6a-i) Online questionnaires: describe if they were validated for online use and apply CHERRIES items to describe how the questionnaires were designed/deployed**		
**6a-ii) Describe whether and how "use" (including intensity of use/dosage) was defined/measured/monitored**		
Yes, as quoted here, "Key outcomes, namely the effectiveness of the smartphone-based intervention, were evaluated by comparing the pre- and post-scores of the Depression, Anxiety, and Stress Scale (DASS-21) [48]. Participants rated the extent to which they experienced depression, anxiety, and stress symptoms (e.g., "I was worried about situations in which I might panic") on a 4-point scale (0 = Did not apply to me at all to 3 = Applied to me very much or most of the time). The Chinese version of the DASS-21 exhibited satisfactory psychometric properties [49]. Cronbach's alpha in the current study was 0.87.".		
**6a-iii) Describe whether, how, and when qualitative feedback from participants was obtained**		
Yes, as quoted here "User activity was measured by numbers of mood journal completed and exercise session taken, as well as average daily session length (in minute).".		
**6b) CONSORT: Any changes to trial outcomes after the trial commenced, with reasons**		
Yes. "Key outcome was measured by the Depression, Anxiety, and Stress Scale (DASS-21; Cronbach's α = 0.87)." But we would like to make it more explicitly in the next round of revision by mentioning that the outcome was assessed via the Neurum.		
**7a) CONSORT: How sample size was determined**		
**7a-i) Describe whether and how expected attrition was taken into account when calculating the sample size**		
**7b) CONSORT: When applicable, explanation of any interim analyses and stopping guidelines**		
Yes, pre-specified primary and secondary outome measures were reported in the manuscript, and in the pre-register too (registration reference: osf. io/z829u).		
**8a) CONSORT: Method used to generate the random allocation sequence**		
Yes, no changes to trial outcomes were made.		
**8b) CONSORT: Type of randomisation; details of any restriction (such as blocking and block size)**		
No, this was not relevant for the study as it involved only healthy, adult participants, no interium analyses were planned. Relatedly, no stopping guidelines were pre-determined, but possible for adverse events, as quoted here, "As part of the protocol, a referral mechanism was established, such that users who score past a certain threshold in their levels of distress are automatically referred to our crisis management protocol, which involves the algorithm providing the user with immediate support via distress reduction modules, crisis hotlines, as well as face to face care delivered by a licensed, on-board clinical psychologist.". Authors would like to clarify that no adverse events were reported.		
**9) CONSORT: Mechanism used to implement the random allocation sequence (such as sequentially numbered containers), describing any steps taken to conceal the sequence until interventions were assigned**		
Yes, as quoted here, "Participants were then randomly allocated at a 1:1 ration to the two groups. A computer algorism was employed for the simple random assignment such that a numerical value between 0 and 1 was assigned to each participant, those who received a value smaller than 0.5 was assigned to the control group, otherwise assigned to the intervention group.".		
**10) CONSORT: Who generated the random allocation sequence, who enrolled participants, and who assigned participants to interventions**		
Yes, a simple randomization procedure was adopted, see subitem 8a.		
**11a) CONSORT: Blinding - If done, who was blinded after assignment to interventions (for example, participants, care providers, those assessing outcomes) and how**		
**11a-i) Specify who was blinded, and who wasn't**		
**11a-ii) Discuss e.g., whether participants knew which intervention was the "intervention of interest" and which one was the "comparator"**		
Yes, as quoted here, "Double-blindness was achieved such that a research assistant who remained unaware of the study design was in charge of the participant assignment, while participants received a unique study ID in a singular format that revealed no group information, and therefore, remained unaware of the grouping.".		
**11b) CONSORT: If relevant, description of the similarity of interventions**		
Yes, as quoted here, "A computer algorism was employed for the simple random assignment such that a numerical value between 0 and 1 was assigned to each participant, those who received a value smaller than 0.5 was assigned to the control group, otherwise assigned to the intervention group.". The excel list which contains the simple randomization results was kept offline in a staff-access only laptop in authors' lab.		
**12a) CONSORT: Statistical methods used to compare groups for primary and secondary outcomes**		
Yes, reference to the full trial was reported.		
**12a-i) Imputation techniques to deal with attrition / missing values**		
**12b) CONSORT: Methods for additional analyses, such as subgroup analyses and adjusted analyses**		
Yes, there was no placebo or sham intervention involved.		
RESULTS		

**13a) CONSORT: For each group, the numbers of participants who were randomly assigned, received intended treatment, and were analysed for the primary outcome**		
Reporting technical details that were overlooked in previous writting.		
**13b) CONSORT: For each group, losses and exclusions after randomisation, together with reasons**		
Yes, as quoted here, "A set of supplementary models including gender, education, and marital status as covariates found similar patterns (FCondition(1)s ≤ 1.601, Ps ≥ .208, η2 ≤ 0.011; FTime(1)s ≥ 2.933, Ps ≤ .086, η2 ≥ 0.020; FTime ✕ Condition(1)s ≥ 19.286, Ps ≤ .001, η2 ≥ 0.117). Another set of supplementary models using DASS-21 dimensional categories to examine the change of severity levels revealed that the control group ascended while the intervention group descended in symptom severity (χ^2s ≥ 8.605, Ps ≤ .018; see Table 4). Finally, and surprisingly, another set of multiple linear regressions revealed that none of exercise session number, mood journal number, or average daily session length predicted the post-intervention DASS-21 scores (see Table 5). ".		
**13b-i) Attrition diagram**		
**14a) CONSORT: Dates defining the periods of recruitment and follow-up**		
Yes, as quoted here, "A priori power analysis ...... a total number of 196 participants was needed to achieve a power of .80. Assuming an attrition rate of 25%, a sample of 262 participants were determined prior to data collection. ......Participant recruitment started in September 2019, Hong Kong. However, due to local social unrest and the Covid-19 pandemic, few participants signed up within the planned one-month period. Therefore, under all authors' approval and the funding agency, the recruitment process was prolonged until the deemed sample size would be achieved. The recruitment process lasted for over a year and was finally terminated by the end of October 2020, when nearly no more participant signed up within a month .In total, 201 working adults were recruited from local companies...... There were significantly more participants dropped from the intervention group (n = 21) than from the control group (n = 2; □2(1) = 18.259, P < .001; overall attrition rate = 11.443%), resulting in 81 participants in the intervention group and 97 in the control group. Considering the intensive EMA/I procedures, the differential attrition rates between the two conditions were not surprising. More importantly, the final sample consisted of 178 working adults and was still sufficient in statistical power.". Also see figure 1 for participant flowchart.		
**14a-i) Indicate if critical "secular events" fell into the study period**		
**14b) CONSORT: Why the trial ended or was stopped (early)**		
Yes, please refer to our answer in subitem 13a.		
**15) CONSORT: A table showing baseline demographic and clinical characteristics for each group**		
Yes, as quoted here, "Participant recruitment started in September 2019, Hong Kong. However, due to local social unrest and the Covid-19 pandemic, few participants signed up within the planned one-month period. Therefore, under all authors' approval and the funding agency, the recruitment process was prolonged until the deemed sample size would be achieved. The recruitment process lasted for over a year and was finally terminated by the end of October 2020, when nearly no more participant signed up within a month".		
**15-i) Report demographics associated with digital divide issues**		
**16a) CONSORT: For each group, number of participants (denominator) included in each analysis and whether the analysis was by original assigned groups**		
**16-i) Report multiple "denominators" and provide definitions**		
**16-ii) Primary analysis should be intent-to-treat**		
Yes, for the control group, n = 97, and for the intervention group, n = 81. This information was reported throughout the results section whenever needed.		
**17a) CONSORT: For each primary and secondary outcome, results for each group, and the estimated effect size and its precision (such as 95% confidence interval)**		
No, this was not addressed in the manuscript as the trial generallly aligned with the pre-registered protocol.		
**17a-i) Presentation of process outcomes such as metrics of use and intensity of use**		
**17b) CONSORT: For binary outcomes, presentation of both absolute and relative effect sizes is recommended**		
Yes, please refer to Table 1 for demographich characteristics and Table 2 for between group comparison of baseline outcome characteristics.		
**18) CONSORT: Results of any other analyses performed, including subgroup analyses and adjusted analyses, distinguishing pre-specified from exploratory**		
Yes, results for each group and effect sizes along with the precision were reported, for example, as quoted here, "Mixed ANOVAs were applied to compare the pre- and post- scores of the DASS-21 and its sub-scales between groups (see Table 3). For the total score, equal variance between groups was satisfied (Levene statistics ≤ 2.127, ps ≥.147). Results revealed that there was no significant difference between the intervention (M = 21.990) and the control (M = 21.852) groups. But as expected, there was a significant post-intervention decrease (Mpre-intervention = 24.721 and Mpost-intervention = 19.121). More importantly, the above effects were quantified by a significant interaction between time and group (see Figure 3 (a)) such that the DASS-21 total scores decreased for the intervention group (Mpre-intervention = 29.111 and Mpost-intervention = 14.593) but increased for the control group (Mpre-intervention = 20.330 and Mpost-intervention = 23.649).".		
**18-i) Subgroup analysis of comparing only users**		
**19) CONSORT: All important harms or unintended effects in each group**		
Yes, a set of supplementary analysis focused on the change of severity levels (i.e., worsen, no change, vs. improved) of the DASS-21 depression, anxiety, and stress dimensions, and results were reported accordingly, as quoted here, "Another set of supplementary models using DASS-21 dimensional categories to examine the change of severity levels revealed that the control group ascended while the intervention group descended in symptom severity (χ^2s ≥ 8.605, Ps ≤ .018; see Table 4). Finally, and surprisingly, another set of multiple linear regressions revealed that none of exercise session number, mood journal number, or average daily session length predicted the post-intervention DASS-21 scores (see Table 5). ".		
**19-i) Include privacy breaches, technical problems**		
**19-ii) Include qualitative feedback from participants or observations from staff/researchers**		
No, this was not relevant in our study, no technical problems or any other unexpected/unintended incidents were observed, though this information was not explicitly reported in the current version of the manuscript, authors would like to further address this in the review process.		
DISCUSSION		
**20) CONSORT: Trial limitations, addressing sources of potential bias, imprecision, multiplicity of analyses**		
**20-i) Typical limitations in ehealth trials**		
**21) CONSORT: Generalisability (external validity, applicability) of the trial findings**		
**21-i) Generalizability to other populations**		
**21-ii) Discuss if there were elements in the RCT that would be different in a routine application setting**		
Yes, as quoted here, "Last but not least, this study expanded the utility of EMA/I beyond clinical settings to community populations, which broadened the spectrum of the recipients of such digital healthcare services.".		
**22) CONSORT: Interpretation consistent with results, balancing benefits and harms, and considering other relevant evidence**		
**22-i) Restate study questions and summarize the answers suggested by the data, starting with primary outcomes and process outcomes (use)**		
**22-ii) Highlight unanswered new questions, suggest future research**		
Yes, as quoted here, "To the best of our knowledge, this was the first study to examine the efficacy of a smartphone application which incorporated EM/I to reduce workplace stress in Asian populations. Consistent with our hypothesis, results demonstrated that the approach was effective in reducing distress after 4 weeks of intervention, as reflected by both score and intensity level changes, suggesting that the EMA/I approach via the Neurum App could support working adults in real time and in naturalistic settings. ".		
Other information		
**23) CONSORT: Registration number and name of trial registry**		
Yes, two sets of supplementary analyses were reported.		
**24) CONSORT: Where the full trial protocol can be accessed, if available**		
Yes, no adverse events were reported, and no harms/unintended effects were observed.		

**25) CONSORT: Sources of funding and other support (such as supply of drugs), role of funders**		
Yes, reference to the trial registry was included.		
**X26-i) Comment on ethics committee approval**		
**x26-ii) Outline informed consent procedures**		
No, comments on ethics commitee approval was not included.		
**X26-iii) Safety and security procedures**		
No, this information was missing in our submitted version and we would like to include it during the review process. The informed consent procedure was like, "Informed consent was obtained such that participants were provided with essential introduction of this study, they provided their electronic signature indicating they understood the intervention procedure, risks and benefits, and confidentiality, upon their first-time log-in to Neurum.".		
**X27-i) State the relation of the study team towards the system being evaluated**		
